# Incidence and baseline predictors of discontinuation in DRCRnet studies

**DOI:** 10.1038/s41433-026-04853-y

**Published:** 2026-09-11

**Authors:** Klyve N. Otinkorang, Gui-shuang Ying

**Affiliations:** 1 University of Pennsylvania Perelman School of Medicine, Philadelphia, PA USA; 2 Center for Preventive Ophthalmology and Biostatistics, Department of Ophthalmology, University of Pennsylvania Perelman School of Medicine, Philadelphia, PA USA

**Keywords:** Risk factors, Epidemiology

## Abstract

**Objective:**

To evaluate the incidence and baseline predictors of participant discontinuation in Diabetic Retinopathy Clinical Research Retina Network (DRCRnet) studies

**Methods:**

Using the publicly available DRCRnet data, discontinuation rate and its 95% confidence intervals (95% CI) were calculated. Univariable and multivariable logistic regression models were used to assess predictors for participant discontinuation, including demographic, clinical, and study design characteristics, and within-study correlation was accounted for using generalised estimating equations.

**Results:**

Among 7353 participants (mean age 60 years, 48.0% female, 64.1% non-Hispanic (NH)-White, 17.8% NH-Black, 14.2% Hispanic), 1110 participants (15.1%, 95% CI 14.3–15.9%) discontinued study participation over a median 24-month follow-up. Multivariable analysis showed significantly higher risk of discontinuation among Hispanic (aOR 1.73, 95% CI 1.38–2.18) and NH-Black (aOR 1.36, 95% CI 1.14–1.62) participants, those with age <50 years (aOR 1.46, 95% CI 1.21–1.77), 50–59 years (aOR 1.26, 95% CI 1.05–1.51), or ≥70 years (aOR 1.20, 95% CI 1.03–1.39), participants with higher baseline HbA1c (aOR 1.09 per unit increase, 95% CI 1.06–1.12), or baseline blood pressure ≥140/90 mmHg (aOR 1.24, 95% CI 1.09–1.41), and those in studies with follow-up length 24 to <36 months (aOR 1.64, 95% CI 1.02–2.64) or 36 to 48 months (aOR 2.68, 95% CI 1.87–3.84).

**Conclusions:**

Approximately 15% of participants in the eligible DRCRnet studies discontinued participation. Racial and ethnic identity, age, glycaemic and blood pressure control, and study follow-up duration are significant predictors of participant discontinuation and should be considered when designing future studies.

## Introduction

Diabetic retinopathy (DR) is one of the leading causes of preventable blindness [1]. Individuals from racial and ethnic minority groups experience a disproportionate burden of DM and its ocular sequelae, including diabetic macular oedema (DMO), and have been found to be more likely to be lost to follow-up in clinical studies of treatments for DR or DMO [2–8]. Poor patient retention in longitudinal care and lapses in treatment for DR accelerate vision loss and disease progression, widening these disparities [9–14]. Since race and ethnicity are social, not biological, constructs, disparities in DR and DMO outcomes and loss to follow-up reflect structural and social determinants of health [15–18].

Clinical studies have been central to improving our understanding of DR and DMO; yet, participant discontinuation remains a critical challenge [2, 4, 5, 17–20]. Discontinuation reduces statistical power, biases treatment effect estimates, and undermines generalisability [2, 21]. Despite these implications, there are relatively few large sample size analyses of discontinuation rates and associated predictors in DR studies. Identifying predictors for discontinuation may inform the design of future DR studies and strategies to promote participant retention.

The Diabetic Retinopathy Clinical Research Retina Network (DRCRnet), a longstanding multicentre research network investigating retinal diseases, has conducted many pivotal clinical studies for evaluating the efficacy of various treatments in DR and DMO [22, 23]. Leveraging its extensive, harmonised datasets provides a unique opportunity to quantify discontinuation rates and identify associated risk factors. Using publicly available DRCRnet datasets, we assessed discontinuation rates and comprehensively evaluated baseline predictors for discontinuation, including demographic, clinical, and study design characteristics. We hypothesised that demographic and clinical factors can predict discontinuation in line with observations in clinical settings and other research areas [2–5, 17, 24–27].

## Methods

### Ethics statement

This study adhered to the principles of the Declaration of Helsinki. It involved secondary analysis of publicly available de-identified data from the DRCR.net website (https://public.jaeb.org/drcrnet/stdy). The requirement for informed consent was waived by the University of Pennsylvania Institutional Review Board.

### Eligible studies

Our secondary data analysis required individual-level data. We included DRCRnet studies (*N* = 19) conducted between 2003 and 2020 that had individual-level data publicly available at the DRCR.net website at the time of data acquisition (October 1, 2025). We excluded studies that were ongoing (6), had less than one-week follow-up (3), lacked publicly available data (5), or did not have information about participant study completion or mortality during follow-up periods (5). Two studies investigating vitreomacular traction were excluded because they included individuals who did not have DM.

### Outcome measure: study discontinuation

Study discontinuation was defined as a participant exiting from a study before its completion for any reason other than death. Participants who died prior to study completion were excluded from analysis of discontinuation.

### Candidate risk factors for study discontinuation

Participants had a documented diagnosis of type 1, type 2, or unknown DM. Those with unknown type were excluded from the analysis. Participants self-identified their race and ethnicity at the time of enrolment. Haemoglobin A1c (HbA1c) level was measured at baseline or recorded from electronic medical records if a measurement was available within 3 months prior to enrolment. Hyperlipidaemia, hypercholesterolaemia, and hypertriglyceridemia statuses were self-reported or documented from participants’ medical records at the time of enrolment and recorded as “yes”, “no”, or “unknown”. Participants who were recorded as having hyperlipidaemia, hypercholesterolaemia, or hypertriglyceridemia were grouped as having dyslipidaemia (DLD). DLD data were unavailable for a substantial proportion of participants because this information was not consistently collected across all included studies. Blood pressure (BP) was measured at baseline and used in this analysis to approximate BP control independent of hypertension diagnosis. Participants were classified into one-eye or two-eye groups based on whether one or both eyes of a participant were included as study eyes for assessing the study outcome. Participants underwent a visual acuity (VA) assessment at baseline using Early Treatment Diabetic Retinopathy Study (ETDRS) or Snellen scores. The R package *eye* was used to convert ETDRS scores to Snellen scores [28]. For participants with two study eyes, the study eye with a worse Snellen score represented the participant’s VA in risk factor analyses.

Based on their disease of interest, studies were grouped as DR, DMO, or DM. Follow-up length for primary outcome measure of the study was categorised as <6 months, ≥6 months to <24 months, ≥24 months to <36 months, or ≥36 months to 48 months based on each study’s original design.

### Statistical analysis

Baseline demographic and clinical characteristics of the participants were summarised using mean and standard deviation (SD) for continuous measures and count (%) for categorical measures. We calculated the discontinuation rate and its 95% confidence interval (95% CI) among all eligible participants. Risk factors for discontinuation were analysed using univariable logistic regression models followed by a multivariable logistic regression model. The potential risk factors included age at enrolment, sex, self-identified race and ethnicity, baseline clinical characteristics (DM type, HbA1c, DLD status, BP measurement, visual acuity score), number of study eyes, study disease of interest, and required follow-up length. Risk factors with *p* < 0.05 from the univariable analysis were included in the initial multivariable logistic regression model, which underwent backward variable selection by only keeping statistically significant risk factors in the final multivariable logistic regression. The unadjusted odds ratios (OR) and adjusted odds ratios (aOR) from univariable and multivariable logistic regression models, respectively, and their 95% CIs were reported. In all logistic regression models, generalised estimating equations was applied to account for within-study correlation among participants from the same study. All statistical analyses were conducted using STATA SE version 18.0 (College Station, TX) with a two-sided *p* < 0.05 considered statistically significant.

## Results

Our study included 7770 participants from 19 DRCRnet studies conducted between 2003 and 2020. After excluding 370 (4.8%) participants who died before study completion and 47 (0.6%) with an unknown DM type, 7353 (94.6%) participants were included in the analysis. Table 1 summarises the demographic and clinical characteristics of included study participants.

**Table 1:** Description of participants in the included DRCRnet studies (*N* = 7353).

Participant characteristics	
**Age at enrolment (Years):** Mean (SD)	60.2 (11.7)
**Sex:** n (%)	
Male	3822 (52.0%)
Female	3531 (48.0%)
**Race and Ethnicity:** n (%)	
Hispanic	1041 (14.2%)
Non-Hispanic Asian	141 (1.9%)
Non-Hispanic Black	1311 (17.8%)
Non-Hispanic White	4711 (64.1%)
Other or Unknown	149 (2.0%)
**Type of diabetes:** n (%)	
Type 1	855 (11.6%)
Type 2	6498 (88.4%)
**HbA1c (%): Mean (SD)**	8.1 (1.8)
**Baseline Blood Pressure:** n (%)	
Normal	1151 (15.7%)
Elevated	907 (12.3%)
Stage 1 Hypertension	1840 (25.0%)
Stage 2 Hypertension	3084 (41.9%)
Unknown	371 (5.0%)
**Dyslipidaemia:** n (%)	
Yes	2600 (35.4%)
No	1095 (14.9%)
Unknown	3658 (49.7%)
**Number of study eyes:** n (%)	
One eye	4629 (63.0%)
Two eyes	2724 (37.0%)
**Study Eye Snellen score:** n (%)	
20/20 or better	1,331 (18.1%)
20/25 to 20/32	1822 (24.8%)
20/40 to 20/80	2596 (35.3%)
20/100 to 20/200	791 (10.8%)
Worse than 20/200	622 (8.4%)
Unknown	191 (2.6%)
**Disease studied**, n (%)	
Diabetes Mellitus	1770 (24.1%)
Diabetic Macular Oedema	3923 (53.4%)
Diabetic Retinopathy	1660 (22.6%)
**Length of follow-up (months)**	
Median (Q1, Q3)	24 (24, 36)
Min, Max	3.7, 48
<6	1145 (15.6%)
6 to <24	726 (9.9%)
24 to <36	3667 (49.9%)
36–48	1815 (24.7%)

Among 7353 eligible participants, 1110 (15.1%, 95% CI 14.3–15.9%) participants discontinued participation. Discontinuation rate varied across DRCRnet studies, ranging from 1.4 to 31.5% with a median (inter-quartile range) discontinuation rate of 10.5% (6.1–21.4%) across 19 included studies. The rate of discontinuation was highest among Hispanic (21.5%) and lowest among NH-White (12.6%) participants.

In univariable analysis (Table 2), Hispanic (OR 1.90, 95% CI 1.52–2.38), NH-Asian (OR 1.88, 95% CI 1.14–3.08), and NH-Black (OR 1.52, 95% CI 1.23–1.88) participants had higher risk of discontinuation than NH-White participants. Participants with age <50 years (OR 1.62, 95% CI 1.28–2.06) or 50–59 years (OR 1.32, 95% CI 1.08–1.62) also had higher risk of discontinuation than participants aged 60–69 years. Baseline BP in the stage 2 hypertension range (OR 1.27, 95% CI 1.03–1.57), higher HbA1c levels (OR 1.14 per unit increase, 95% CI 1.11–1.17), and having two study eyes (OR 1.64, 95% CI 1.11–2.41) were associated with higher risk of discontinuation. The Protocol M trial [29] was the only study with DM as its disease of interest. Participation in Protocol M (OR 2.34, 95% CI 1.52–3.61) or studies with longer follow-up conferred higher risk of discontinuation, with OR of 1.99 (95% CI 1.09–3.60) for follow-up length 6 to <24 months, 2.67 (95% CI 1.44–4.94) for 24 to <36 months, and 3.31 (95% CI 2.48–3.60) for 36 months to 48 months. In contrast, sex, DM type, DLD status, and baseline Snellen score were not significantly associated with discontinuation.

**Table 2:** Univariable analysis for factors associated with study discontinuation (*N* = 7353).

	Number of participants	Discontinuation (%)	Unadjusted odds ratio (95% CI)	*P* value
**Age at enrolment (Years)**				
<50	1202	230 (19.1%)	1.62 (1.28–2.06)	<0.001
50– 59	2091	338 (16.2%)	1.32 (1.08–1.62)	0.007
60–69	2577	328 (12.7%)	Reference	
≥70	1483	214 (14.4%)	1.16 (0.99–1.36)	0.07
**Sex**				
Male	3822	550 (14.4%)	Reference	
Female	3531	560 (15.9%)	1.12 (0.97–1.30)	0.13
**Race and Ethnicity**				
Hispanic	1041	224 (21.5%)	1.90 (1.52–2.38)	<0.001
Non-Hispanic Asian	141	30 (21.3%)	1.88 (1.14–3.08)	0.06
Non-Hispanic Black	1311	236 (18.0%)	1.52 (1.23–1.88)	<0.001
Non-Hispanic White	4711	593 (12.6%)	Reference	
Other or Unknown	149	27 (18.1%)	1.54 (0.94–2.51)	0.11
**Type of Diabetes**				
Type 1	855	125 (14.6%)	1.07 (0.94–1.21)	0.34
Type 2	6,498	985 (15.2%)	Reference	
**Baseline Blood Pressure**				
Normal	1151	156 (13.6%)	Reference	
Elevated	907	130 (14.3%)	1.07 (0.90–1.27)	0.47
Stage 1 Hypertension	1840	285 (15.5%)	1.17 (0.98–1.40)	0.08
Stage 2 Hypertension	3084	513 (16.6%)	1.27 (1.03–1.57)	0.03
Unknown	371	26 (7.0%)	0.48 (0.19–1.23)	0.13
**Dyslipidaemia**				
Yes	2600	350 (13.5%)	0.90 (0.69–1.16)	0.40
No	1095	162 (14.8%)	Reference	
Unknown	3658	598 (16.3%)	1.13 (0.57–2.24)	0.74
**Number of Study Eyes**				
One	4629	587 (12.7%)	Reference	
Two	2724	523 (19.2%)	1.64 (1.11–2.41)	0.01
**Study Eye Snellen Score**				
20/20 or better	1331	207 (15.6%)	Reference	
20/25 to 20/32	1822	238 (13.1%)	0.82 (0.62–1.08)	0.15
20/40 to 20/80	2596	402 (15.5%)	0.99 (0.65–1.53)	0.98
20/100 to 20/200	791	147 (18.6%)	1.24 (0.67–2.28)	0.49
Worse than 20/200	622	108 (17.4%)	1.14 (0.68–1.93)	0.62
Unknown	191	8 (4.2%)	0.24 (0.01–3.80)	0.31
**HbA1c** (per 1% increase)	6,674	1,053 (15.8%)	1.14 (1.11–1.17)	<0.001
**Disease Studied**				
Diabetic Macular Oedema	3923	417 (10.6%)	Reference	
Diabetic Retinopathy	1660	307 (18.5%)	1.91 (0.99–3.66)	0.052
Diabetes Mellitus	1770	386 (21.8%)	2.34 (1.52–3.61)	<0.001
**Length of Follow-up (Months)**				
<6	1145	77 (6.7%)	Reference	
6 to <24	726	91 (12.5%)	1.99 (1.09–3.60)	0.02
24 to <36	3667	592 (16.1%)	2.67 (1.44–4.94)	0.002
36 to 48	1815	350 (19.3%)	3.31 (2.48–3.60)	<0.001

In multivariable analysis of 6674 participants, after excluding 679 individuals with missing HbA1c data (Table 3), racial and ethnic identity, age at enrolment, HbA1c level, baseline BP, study disease of interest, and required follow-up period length remained significant predictors of discontinuation. Hispanic (aOR 1.73, 95% CI 1.38–2.18) and NH-Black (aOR 1.36, 95% CI 1.14–1.62) racial and ethnic identity and baseline age <50 years (aOR 1.46, 95% CI 1.21–1.77), 50–59 years (aOR 1.26, 95% CI 1.05–1.51), or ≥70 years (aOR 1.20, 95% CI 1.03–1.39) were independently associated with higher risk of discontinuation. Higher baseline HbA1c levels (aOR 1.09 per unit increase, 95% CI 1.06–1.12) or stage 2 hypertension range baseline BP (aOR 1.24, 95% CI 1.09–1.41) were associated with higher risk of discontinuation. Participation in Protocol M (aOR 2.27, 95% CI 1.55–3.32) was associated with higher risk of discontinuation. Longer follow-up length was associated with higher risk of discontinuation for studies with length of follow-up 24 to <36 months (aOR 1.64, 95% CI 1.02–2.64) and 36 months to 48 months (aOR 2.68, 95% CI 1.87–3.84). However, studies with follow-up length of 6 to <24 months were no longer independently associated with discontinuation after multivariable adjustment (aOR 1.58, 95% CI 0.83–3.02).

**Table 3:** Multivariable analysis for factors associated with study discontinuation (*N *= 6674).

	Number of participants	Discontinuation (%)	Adjusted odds ratio (95% CI)*	*P* value
**Race and Ethnicity**				
Hispanic	957	214 (22.4%)	1.73 (1.38–2.18)	<0.001
Non-Hispanic Asian	130	27 (20.8%)	1.56 (0.92-2.64)	0.10
Non-Hispanic Black	1204	230 (19.1%)	1.36 (1.14–1.62)	0.001
Non-Hispanic White	4250	558 (13.1%)	Reference	
Other or Unknown	133	24 (18.1%)	1.41 (0.88–2.26)	0.15
**Age at enrolment (Years)**				
<50	1076	222 (20.6%)	1.46 (1.21–1.77)	<0.001
50–59	1898	316 (16.7%)	1.26 (1.05–1.51)	0.01
60–69	2358	310 (13.2%)	Reference	
≥70	1342	205 (15.3%)	1.20 (1.03–1.39)	0.02
**HbA1c (%)**	6674	1,053 (15.8%)	1.09 (1.06–1.12)	<0.001
**Baseline Blood Pressure**				
Normal	1038	154 (14.8%)	Reference	
Elevated	795	123 (15.5%)	1.09 (0.91–1.30)	0.34
Stage 1 Hypertension	1690	274 (16.2%)	1.14 (0.98–1.33)	0.10
Stage 2 Hypertension	2869	488 (17.0%)	1.24 (1.09–1.41)	0.001
Unknown	282	14 (5.0%)	0.59 (0.34–1.02)	0.06
**Disease Studied**				
Diabetic Macular Oedema	3324	381 (11.5%)	Reference	
Diabetes Mellitus	1747	380 (21.8%)	2.27 (1.55–3.32)	<0.001
Diabetic Retinopathy	1603	292 (18.2%)	1.50 (0.96–2.33)	0.07
**Length of follow-up (Months)**				
<6	992	62 (6.3%)	Reference	
6 to <24	693	83 (12.0%)	1.58 (0.83–3.02)	0.16
24 to <36	3224	577 (17.9%)	1.64 (1.02–2.64)	0.04
36–48	1765	331 (18.8%)	2.68 (1.87–3.84)	<0.001

*Adjusted odds ratios and 95% confidence intervals were estimated from multivariable logistic regression model adjusted for race and ethnicity, haemoglobin A1c (HbA1c), baseline blood pressure measurement, disease studied, and study length of follow-up. Total of 679 subjects were excluded from the multivariable analysis due to missing data in HbA1c.

## Discussion

In this study, we found approximately 15% of participants in the eligible DRCRnet studies discontinued participation, similar to 12.44% rate in anti-VEGF clinical trials of retinal diseases reported by Rosenblatt [27]. Demographic, clinical, and study design characteristics were identified as significant predictors of participant discontinuation in DRCRnet studies, highlighting factors to consider to improve participant retention in future studies.

Hispanic and NH-Black participants had higher risk of discontinuation than NH-White participants. Previous cohort studies investigating participant discontinuation risk factors in patients receiving longitudinal care for DR and DMO had similar results [3–5, 20]. Black and Hispanic patients have been identified as being more susceptible to discontinuation in longitudinal care for other ophthalmologic conditions, such as glaucoma and age-related macular degeneration [30, 31]. These disparities are also present in research in other fields, including cardiovascular and oncological clinical trials, suggesting poor retention of Hispanic and NH-Black individuals is not limited to DRCRnet studies and may reflect pervasive systemic barriers in the clinical and research domains of multiple medical fields [4, 5, 24, 32].

Race and ethnicity should not be interpreted as causal biological predictors of study discontinuation. Rather, these variables likely serve as markers of structural and social factors that may influence research participation and retention. Several factors have been proposed to contribute to underrepresentation and poorer retention of racial and ethnic groups in clinical research, such as distrust of researchers, concerns about lost wages and financial burden, language barriers, limited transportation access and travel burden, inadequate family or social support, and housing instability or lack of reliable contact information [33–37]. Strategies implemented to combat these disparities often target participants on individual, institutional, community, and political levels and address social determinants of health [38]. For future studies involving underrepresented populations, addressing these disparities may require educating patients about the study’s significance and required time commitments, offering appointment scheduling flexibility, assisting with transportation payments, collaborating with the local community and patient advocacy groups to build trust, and training research staff to identify and address biases and utilise culturally competent communication [38–41]. Implementing these steps across clinical sites may increase participants’ perceived value of their contribution and decrease external barriers to their involvement; thus, promoting study completion [39, 42].

We found a significant non-monotone association between age of participants and discontinuation. Participants who were ≤59 years or ≥70 years at the time of enrolment had higher odds of discontinuation compared to those with age of 60–69 years, concordant with findings from a study of anti-VEGF and panretinal photocoagulation in proliferative DR [4]. Conflicts with work and childcare obligations, travel time to appointments, and appointment duration can contribute to discontinuation [42, 43]. Younger adults tend to allocate more time to work and childcare, increasing their susceptibility to these challenges. Older adults typically have more leisure time, but experience an increased risk of multimorbidity and greater reliance on others for assistance with activities of daily living and transportation, which are discontinuation risk factors among post-retirement age adults [43–46]. We speculate that adults aged 60 to 69 may be generally healthier than those 70 years or older, while having fewer obligations than those 59 years or younger. Thus, their autonomy, health state, and higher amount of free time may allow for more consistent study participation.

Higher A1c and baseline blood pressure in the stage 2 hypertension range were independently associated with increased risk of discontinuation. Higher A1c and hypertension can reflect irregular health access, poor medication adherence, and treatment failure, among other issues [47–50]. Comorbidities in the setting of DR and DMO have also been linked to higher healthcare visit burden and noncompliance in research studies [2, 51, 52]. We did not observe an association between DLD status and discontinuation. However, almost half of participants had an unknown DLD status, potentially obscuring the relationship between discontinuation and DLD status.

Longer required follow-up lengths were associated with discontinuation, which may be attributable to increasing opportunity costs of attending study-related appointments [42]. Although studies requiring 6 to <24 months of follow-up had a significant two-fold higher discontinuation risk in the univariable analysis, their association was attenuated and no longer statistically significant after adjustment for other covariates, suggesting that participant or study characteristics may partially explain the observed relationship. Protocol M involved the intervention group having more frequent follow-up visits, potentially creating a between-group difference in follow-up burden and increasing risk of discontinuation. This should be further evaluated in future studies.

Our analysis has several limitations. Of the 32 currently completed DRCRnet studies, only 19 (57.6%) studies met our analysis eligibility, limiting the generalisability of our findings. Variables were selected for the final multivariable model using backward selection with a significance threshold of *p* < 0.05, meaning the final model may be susceptible to overfitting. Our analysis did not assess reasons for study discontinuation because such data were not always available. Since we focused on baseline predictors, we did not investigate the effect of longitudinal developments, such as adverse events, changes in visual acuity, and worsening health status, which have been associated with discontinuation in other DR studies [2, 11, 18]. We also could not extensively evaluate social determinants of health as risk factors of study discontinuation because this information was not captured in most studies. Further studies are needed to evaluate how study discontinuation was associated with socioeconomic status, including educational attainment, annual income, insurance status, zip code, and geographic region, and other hurdles to study participation, such as distance from home to study site, primary language or language barriers, health literacy, and access to regular transportation, known contributors to study discontinuation [2, 4, 17, 19]. Future DRCRnet studies may benefit from prospectively collecting such information to more comprehensively characterise study populations and better understand factors associated with study discontinuation. We were unable to comprehensively evaluate the association between comorbidities and study discontinuation because completeness of comorbidity data varied across studies. Other studies have identified an additive effect of comorbidities on the likelihood of discontinuation with Green et al. observing an association between discontinuation and having five or more comorbidities among patients receiving treatment for proliferative DR [18]. DRCRnet study participants often experienced multiple comorbidities, but we studied only two conditions with an established relationship with DR and DMO: DLD and hypertension [47, 48, 53]. Future studies should involve more detailed analyses of the factors contributing to study discontinuation.

In conclusion, approximately 15% of participants in the eligible DRCRnet studies discontinued study participation. Racial and ethnic identity, age, baseline glycaemic and blood pressure control, and required length of follow-up are significant predictors of study discontinuation. These findings may help identify participants who could benefit from targeted retention efforts and additional support. Incorporating such strategies into the design and conduct of future studies may help address barriers to study participation, reduce discontinuation, enhance study validity, and promote equity in clinical research.

## Summary

### What was known before:

- Clinical studies of diabetic retinopathy and diabetic macular oedema often face challenges with participant retention, especially among racial and ethnic minority groups, but there are relatively few large sample-size analyses of discontinuation rates and associated predictors.

### What this study adds:

- This study revealed age has a non-monotone association with participant discontinuation.
- Racial and ethnic identity, baseline blood pressure and HbA1c, and longer required follow-up are also significant predictors of discontinuation.

## Data Availability

The DRCRnet study data is available at the DRCR Retina Network public website.
